# Effect of a Nutrition Supplement and Physical Activity Program on Pneumonia and Walking Capacity in Chilean Older People: A Factorial Cluster Randomized Trial

**DOI:** 10.1371/journal.pmed.1001023

**Published:** 2011-04-19

**Authors:** Alan D. Dangour, Cecilia Albala, Elizabeth Allen, Emily Grundy, Damian G. Walker, Cristian Aedo, Hugo Sanchez, Olivia Fletcher, Diana Elbourne, Ricardo Uauy

**Affiliations:** 1 Department of Nutrition and Public Health Intervention Research, Faculty of Epidemiology and Population Health, London School of Hygiene & Tropical Medicine, London, United Kingdom; 2 Instituto de Nutrición y Tecnología de los Alimentos, University of Chile, Santiago, Chile; 3 Department of Medical Statistics, Faculty of Epidemiology and Population Health, London School of Hygiene & Tropical Medicine, London, United Kingdom; 4 Department of Population Studies, Faculty of Epidemiology and Population Health, London School of Hygiene & Tropical Medicine, London, United Kingdom; 5 Department of International Health, Bloomberg School of Public Health, Johns Hopkins University, Baltimore, United States of America; 6 Human Development Department, Latin America and the Caribbean Region, The World Bank, Washington (D.C.), United States of America; University of Cambridge, United Kingdom

## Abstract

**Background:**

Ageing is associated with increased risk of poor health and functional decline. Uncertainties about the health-related benefits of nutrition and physical activity for older people have precluded their widespread implementation. We investigated the effectiveness and cost-effectiveness of a national nutritional supplementation program and/or a physical activity intervention among older people in Chile.

**Methods and Findings:**

We conducted a cluster randomized factorial trial among low to middle socioeconomic status adults aged 65–67.9 years living in Santiago, Chile. We randomized 28 clusters (health centers) into the study and recruited 2,799 individuals in 2005 (∼100 per cluster). The interventions were a daily micronutrient-rich nutritional supplement, or two 1-hour physical activity classes per week, or both interventions, or neither, for 24 months. The primary outcomes, assessed blind to allocation, were incidence of pneumonia over 24 months, and physical function assessed by walking capacity 24 months after enrolment. Adherence was good for the nutritional supplement (∼75%), and moderate for the physical activity intervention (∼43%). Over 24 months the incidence rate of pneumonia did not differ between intervention and control clusters (32.5 versus 32.6 per 1,000 person years respectively; risk ratio = 1.00; 95% confidence interval 0.61–1.63; *p* = 0.99). In intention-to-treat analysis, after 24 months there was a significant difference in walking capacity between the intervention and control clusters (mean difference 33.8 meters; 95% confidence interval 13.9–53.8; *p* = 0.001). The overall cost of the physical activity intervention over 24 months was US$164/participant; equivalent to US$4.84/extra meter walked. The number of falls and fractures was balanced across physical activity intervention arms and no serious adverse events were reported for either intervention.

**Conclusions:**

Chile's nutritional supplementation program for older people is not effective in reducing the incidence of pneumonia. This trial suggests that the provision of locally accessible physical activity classes in a transition economy population can be a cost-effective means of enhancing physical function in later life.

**Trial registration:**

Current Controlled Trials ISRCTN 48153354

*Please see later in the article for the Editors' Summary*

## Abstract 2

Alan Dangour and colleagues report results from the CENEX (Cost-effectiveness Evaluation of a Nutritional supplement and EXercise program for older people) trial, which evaluates a nutritional and exercise program aiming to prevent pneumonia and physical decline in Chilean people.

## Editors' Summary

**Background:**

By 2050, about a quarter of the world's population will be aged 60 years or over, with Asia and Latin America experiencing the most dramatic increases in the proportion of older people. For example, in Chile, which has recently undergone rapid demographic transition, the proportion of the population aged 60 years or over has increased from 8% to 12% over the past 25 years.

Current global policy initiatives that promote healthy ageing include an emphasis on adequate nutrient intakes, as longitudinal studies (conducted in high-income countries) suggest that achieving nutritional sufficiency and maintaining moderate levels of physical activity both decrease risk of mortality by preserving immune function and lean body mass and so reduce the numerous risk factors for disability and chronic disease in later life. Such interventions may also decrease the risk of infection, particularly pneumonia, a common cause of death in older people. However, older people in low- and middle-income countries frequently have diets with insufficient calories (energy) and/or micronutrients.

**Why Was This Study Done?:**

Currently, there is no high-quality evidence to support the benefits of improved nutrition and increased physical activity levels from low-income or transition economies, where the ongoing demographic trends suggest that the needs are greatest. National policies aimed at preserving health and function in older people with interventions such as cash-transfers and provision of “food baskets” are often used in Latin American countries, such as Chile, but are rarely formally evaluated. Therefore, the purpose of this study (the Cost-effectiveness Evaluation of a Nutritional supplement and EXercise program for older people—CENEX) was to evaluate Chile's national nutritional supplementation program and/or physical exercise, to investigate whether this program prevented pneumonia and physical functional decline in older people in Santiago, and also to investigate whether these interventions were cost-effective.

**What Did the Researchers Do and Find?:**

The researchers randomly allocated 28 participating health centers in Santiago, Chile, into one of four arms: (1) nutritional supplementation; (2) nutritional supplementation+physical activity; (3) physical activity alone; (4) control. From May to December 2005, 2,799 eligible adults aged 65–67.9 years and living in low to middle socioeconomic circumstances, who attended each health center, were recruited into the study and received the allocated intervention—daily micronutrient-rich nutritional supplement, or two 1-hour physical activity classes per week, or both interventions or neither—for 24 months. The researchers did not know the allocation arm of each patient and over the course of the study assessed the incidence of pneumonia (viral and bacterial as based on diagnosis at the health center or hospital) and physical function was measured by walking capacity (meters walked in 6 minutes). The researchers used administrative records and interviews with staff and patients to estimate the cost-effectiveness of the interventions.

Participant retention in the study was 84%, although only three-quarters of patients receiving the nutritional intervention and less than half (43%) of patients in the physical activity intervention arm adhered to their respective programs. Over 24 months, the incidence rate of pneumonia did not differ between intervention and control groups (32.5 versus 32.6 per 1,000 person years, respectively), but at the end of the study period, there was a significant difference in walking capacity between the intervention and control clusters (mean difference 33.8 meters). The number of falls and fractures in the study arms were similar. The overall costs over 24 months were US$91.00 and US$163.70 per participant for the nutritional supplement and physical activity interventions, respectively. The cost of the physical activity intervention per extra meter walked at 24 months was US$4.84.

**What Do These Findings Mean?:**

The results of this trial suggest that there is little evidence to support the effectiveness of Chile's national nutritional supplementation program in reducing the incidence of pneumonia for 65.0–67.9 year olds. Therefore, given Chile's high burden of infectious and nutrition-related chronic diseases and the associated high health costs, this program should not be considered as a priority preventive public health intervention. However, the provision of locally available physical activity classes to older people could be of clinical benefit, especially in urban settings such as Santiago, although future challenges include increasing the uptake of, and retention to, such programs.

**Additional Information:**

Please access these Web sites via the online version of this summary at http://dx.doi.org/10.1371/journal.pmed.1001023.

- The World Health Organization provides information about the state of health in Chile
- Wikipedia also provides information about health and health care in Chile (please note that Wikipedia is a free online encyclopedia that anyone can edit; available in several languages)

## Introduction

The United Nations estimates that by 2050, individuals aged 60 y or older will represent 22% of the world's population, or about 2 billion people, with the most dramatic increases in Asia and Latin America [1]. Older people especially in low- and middle-income countries frequently consume diets that may be insufficient in terms of calorie content as well as poor in quality in terms of mineral and vitamin (or micronutrient) composition [2]. Current global policy initiatives are designed to promote healthy ageing and include an emphasis on securing adequate nutrient intakes [3]. Achieving nutritional sufficiency [4] and maintaining moderate physical activity [5] have both been demonstrated to decrease the risk of mortality in longitudinal studies. The benefits of adequate nutrition, especially in terms of the micronutrient content of diets, may arise from preserving immune function and lean body mass, both of which decline with ageing [6]–[8], while physical activity reduces numerous risk factors for disability and chronic diseases in later life [9].

Current evidence from the few clinical trials in older people of micronutrient supplementation for the prevention of infection is largely negative [10],[11], but this evidence is derived exclusively from studies conducted in high-income settings (Northern Europe and North America). Evidence on the benefits of resistance training for the maintenance of physical function in later life comes exclusively from high-intensity, short-term interventions [12]. There is currently no high-quality evidence for either of these interventions in low-income or transition economies where the ongoing demographic trends suggest that the needs for such interventions are greatest [1],[2]. This is an important omission from the current evidence base because the impacts in low-income or transition economies of physical activity and nutritional interventions may differ from those in higher income countries, given the differences in baseline population health and in calorie and micronutrient intake sufficiency.

Chile has undergone a rapid demographic transition and the proportion of the population aged ≥60 y has increased from 8% to 12% over the past 25 y. Pneumonia is common among older people in Chile, with an estimated incidence rate among those aged ≥65 y of 73 per 1,000 person years (py), and is the leading cause of death among those over 80 y of age [13]–[15]. Low levels of physical activity are also common, and 96% of the ≥65 y Chilean population are sedentary [16]. Furthermore, vitamin and mineral dietary intake [17] and status [18] are known to be poor among older people in Chile. Since 1998, the government of Chile has distributed micronutrient fortified foods to all individuals ≥70 y, registered at primary health centers and enrolled in the Programme of Complementary Feeding for the Older Population (PACAM). In 2003, stakeholder groups proposed that the national program could be extended to people aged ≥65y and enhanced by the inclusion of a physical activity component [19]. National policies aimed at preserving health and function in older people with interventions, such as cash-transfers [20] and provision of “food baskets” [21], are often used in Latin American countries but are rarely formally evaluated.

The primary aim of this study, the Cost-effectiveness Evaluation of a Nutritional supplement and EXercise program for older people (or CENEX) study, was to evaluate formally whether Chile's national nutritional supplementation program and/or physical exercise prevented pneumonia and functional decline in older people in Santiago, and whether these interventions were cost-effective. There were two primary hypotheses for the CENEX study. First, that provision for 24 mo of a micronutrient-fortified nutritional supplement to adults aged 65.0–67.9 y would decrease the incidence of pneumonia, and second that provision for 24 mo of a community-based, twice-weekly resistance training exercise program to adults aged 65.0–67.9 y would increase walking capacity [22].

## Methods

### Ethics Statement

The study was approved by ethics committees at Instituto de Nutrición y Tecnología de los Alimentos (INTA; University of Chile), Ministry of Health (Government of Chile), and London School of Hygiene & Tropical Medicine (LSHTM; University of London). All study participants provided full informed written consent before being enrolled in the study.

This is a 2×2 factorial cluster randomized controlled trial of the effect of a 2-y nutritional supplementation and/or physical activity program delivered at the community level to eligible individuals aged 65.0–67.9 y (Text S1). The age range was selected as it met the proposal to extend the national program down to the ≥65-y age group and as it allowed individuals at the upper end of the age range to join the existing national program (open to individuals aged ≥70 y) once they had completed 24 mo in the study. Clusters were health centers with more than 400 residents aged 65.0–67.9 y in low-middle socioeconomic status municipalities (average population 127,000 individuals) in the Santiago Metropolitan area. Detailed methods are published [22] and summarized here (Text S2).

### Interventions

#### Nutritional supplement intervention

The intervention took the form of two nutritional products given to participants at health centers in 1-kg bags with calibrated measuring spoons: 1 kg/mo of “Años Dorados,” a vegetable powdered food, and 1 kg/mo of “Bebida Láctea,” a powdered low-lactose milk-based drink (Table S1). Study participants were given full instructions on how to prepare the products by health center auxiliary nurses; the instructions were also printed on the side of the product packets. Participants were advised to consume the recommended daily serving of 50 g of each of these nutritional supplements, which in total provide 50% of micronutrient and 20% of energy requirements for this age group [23],[24]. Adherence was defined a priori as collecting >1 kg/mo of the supplements (i.e., collection of 2 kg in 12 of 24 mo) as systematically documented in the records kept by health center auxiliary nurses.

#### Physical activity intervention

Participants were offered two 1-h supervised physical activity group training sessions per week. Participants were encouraged to walk to sessions, and following a warm-up period, resistance exercises [25] consisted of chair stands, modified squats and step-ups, and arm pull-ups using rubber bands of variable resistance. Following participant requests, a protocol of recreational activities such as dancing and ball games was also included in all physical activity clusters about 6–9 mo into the study. Those unable to attend were provided with specially designed pictorial guides outlining how exercises should be conduct at home. Adherence was defined a priori as registered attendance at a minimum of 24 classes spread over at least 12 mo.

### Primary Outcomes

The primary outcome for the nutritional supplement intervention was incidence of pneumonia during the 24 mo after enrolment, based on health center diagnosed or hospitalized (J10–J12 [viral] and J13–J18 [bacterial] [26]) cases. The primary outcome for the physical activity intervention was walking capacity (meters walked in 6 min) 24 mo after enrolment.

### Secondary Outcomes

The secondary outcomes were: body mass index (BMI); incidence of acute lower respiratory infection (ALRI); self-reported incidence of falls, fractures, and chronic diseases; timed up-and-go (TUG) [27]; physical and functional limitations; depression (15-item Geriatric Depression Scale [GDS-15]) [28]; self-reported general health and well-being (36-item Short-Form health survey [SF-36]) [29] and productive activity in the household and community; blood pressure; anthropometry; and blood indices of cardiovascular disease risk and insulin resistance (subsample) [22]. We present key secondary outcomes; others will be reported in subsequent publications.

### Recruitment

**Figure 1:**
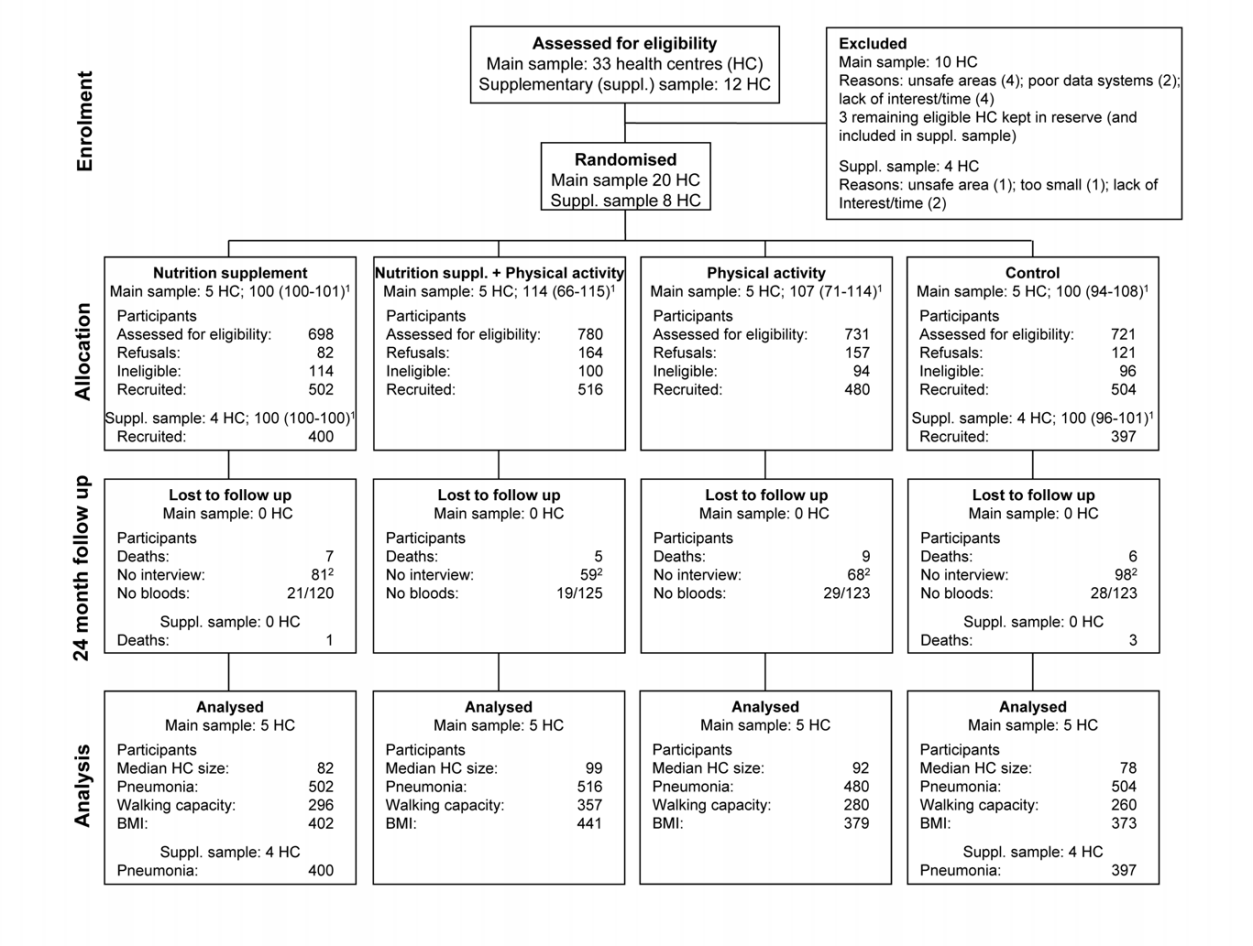
CONSORT flow-chart for CENEX study. ^1^Median (range) health center (HC) sample size. ^2^An additional 12, 11, 24, and 27, did not contribute to the analysis of BMI as either baseline BMI or 24-mo BMI were missing.

At the start of the study, 20 of 33 potential health centers expressed interest. The randomization sequence for study clusters was generated by members of the study team and excluded any outcome assessors. The center names (clusters) were put into a hat. The four treatment arms (nutritional supplementation, nutritional supplementation+physical activity, physical activity, control) were randomly numbered 1–4. As each name was drawn out of the hat by a member of the study team, it was assigned to the next treatment number until each arm contained five clusters. Concerns that lower than expected pneumonia incidence rates would affect the trial's ability to detect an effect of nutritional supplementation on pneumonia incidence resulted in a protocol amendment 4 mo into the study. A further 12 health centers were approached to join the study, eight of which were subsequently randomly assigned by drawing names out of a hat to either the nutritional supplement intervention alone arm (*n* = 4) or the control arm (*n* = 4) (see Figure 1 and Sample Size Calculations). Control arm clusters received neither intervention.

Participants were recruited from May to December 2005. Recruitment was initially from randomly sampled households in health center catchment areas. From July 2005 recruitment was based on health center registries [22]. After exclusions (unable to walk unaided, seeking medical advice for unplanned 3-kg weight loss over 3 mo, planning to move house within 3 mo, already enrolled in national PACAM program or reporting current consumption of PACAM program supplements), remaining potential participants were invited to baseline appointments, provided with detailed information, and screened for cognitive function using a 19-item Mini Mental State Examination (MMSE) [30]. Those with an MMSE score <13 were further assessed using the 11-item Pfeffer screen [31], and those scoring ≥6 were excluded [32]. Remaining individuals who provided written informed consent were enrolled.

### Cost-effectiveness Data

Detailed methods are published [33] and summarized here (Text S3). Estimates of total quantities of resources employed in providing interventions were multiplied by their respective unit prices on the basis of information from administrative records and interviewing staff. Participant costs relating to interventions, i.e., transportation and time, were collected by exit interviews (*n* = 94 for nutritional supplementation intervention; *n* = 93 for physical activity intervention). Participant time was valued using the 2005 national minimum wage (127,500 Chilean pesos). Costs were converted from Chilean pesos to US dollars using 2005 mean exchange rate of US$1 = 560 Chilean pesos.

### Sample Size Calculations

#### Pneumonia

The effect size (33% reduction in community-based pneumonia incidence) was based on the observed difference in pneumonia rates between high- and low-income groups in Santiago [13], and an estimated community-based incidence of 72–90 per 1,000 py. Significantly lower overall incidence rates derived from the first 4 mo of the study (25 cases per 1,000 py) resulted in an increase in the number of clusters from 20 to 28 for this outcome only, and recruitment of 100 participants in each cluster [22]. An intracluster coefficient (ICC) of 0.001 was assumed (consistent with the Aberdeen database [http://www.abdn.ac.uk/hsru/research/delivery/behaviour/methodological-research] of ICCs in primary care). We monitored health center systematic registries of pneumonia cases and related medication prescriptions on a weekly basis, thereby preventing significant loss to follow-up. Furthermore, we collected self-reported incidence of pneumonia at the 12-mo and 24-mo interview.

#### Walking capacity

Mean change, variability, and coefficient of variation (CV) of walking capacity were based on the control arm of a previous study, and the expected impact of the intervention was assumed to be 13%, i.e., half that observed in the earlier trial that intensively supported participants [25]. To detect this effect size with 5% significance, 90% power, and 20% drop-out required ten clusters per arm with 100 participants per cluster.

Power calculations for the primary outcomes assumed no important interactions between the two interventions. This assumption was formally tested and was not rejected for the two primary outcomes pneumonia incidence (*p* = 0.54) and walking capacity (*p* = 0.32). However, interaction between interventions, reflected by change in BMI, was a secondary outcome. The estimated mean BMI, and its variability and CV (10%) were based on the control arm of a previous study [25]. To detect a 0.5-unit mean change in BMI, with *p*<0.05, 90% power and five clusters per arm, required 32 participants per cluster.

Fasting blood indicators of cardiovascular risk and insulin resistance were assessed in a subsample of randomly selected participants. Assuming a CV of 10%, 80% power, 5% significance, and 20% drop-out, a sample of 120 individuals per study arm was sufficient to detect 0.5 SD changes.

### Data Collection

Self-reported baseline data were collected by interview. Anthropometry, blood pressure, TUG, and 6-min walk were assessed at a further appointment. Health center pneumonia cases were identified from health center records based on predefined and verified 2006 national guidelines (Acceso Universal de Garantías Explícitas [AUGE]) [34],[35]. Field staff also reviewed registries of acute respiratory disease units in each health center. Hospitalized cases of pneumonia were abstracted from computerized Ministry of Health databases. Diagnosis of pneumonia or ALRI was made by health center or hospital physicians not involved in the study and blind to the individual's study enrolment.

Participants in the original 20 clusters were re-interviewed after 12 and 24 mo for outcome data. Repeat fasting blood samples were requested at 24 mo. Because of their delayed entry into the study, participants in the eight additional clusters were enrolled for 18 mo (two winter seasons) and interviewed only at baseline with a shorter questionnaire that focused on the pneumonia primary outcome. Pneumonia incidence data were collected for 6 mo after interventions ended in all 28 clusters.

### Data Processing and Statistical Analysis

Data entry and validation checks were conducted at INTA. Analysis was conducted by statisticians at INTA and the LSHTM. The independent Data Monitoring Committee (DMC) assessed on-going compliance and focused on safety; recommendations about stopping for effectiveness were guided by the stringent Peto-Haybittle rule [36],[37]. Primary analyses are intention to treat, further exploratory per-protocol analyses were also conducted. Results are presented as effect sizes with 95% confidence intervals (CIs), accounting for the clustered design [38],[39].

All individuals were counted as “at risk” from their date of entry to the study until they developed pneumonia, died, or completed the study after 2 y (main sample) or 18 mo (supplementary sample). Individuals who died were censored at date of death. Those who developed pneumonia were excluded from the “at risk” group for 14 d after developing pneumonia, after which they re-entered the “at risk” group. Rates in each arm and in each center were calculated as the number of events divided by the person years at risk. The number of occurrences of pneumonia in each study participant showed significant over-dispersion compared to a Poisson distribution; a negative binomial regression model was, therefore, used to estimate rate ratios. The clustered design of the study was taken into account by the use of robust standard errors for estimating the 95% CIs. Both pneumonia and ALRI were considered to be events for the analysis of combined outcomes. All statistical analyses were carried out in STATA version 10 and all statistical tests were two-sided. Cost and effectiveness data were used to derive incremental cost-effectiveness ratios [33].

## Results

The flow of clusters and participants through the trial is shown in Figure 1. In the original 20 clusters, the proportion of eligible participants recruited to the study was highest in the nutritional supplementation only arm and retention at 24 mo was lowest in the control arm. Baseline characteristics of clusters and participants were similar between trial arms (Table 1). (Baseline information for the 28 clusters in the nutritional supplement versus no nutritional supplement comparison is shown in Table S2 and baseline information for the 20 clusters in the physical activity versus no physical activity comparison is shown in Table S3.)

**Table 1:** Baseline characteristics of clusters and individuals by trial arm in the 20 original clusters and the eight supplementary clusters.

Variable	Original Sample Trial Arm				Supplementary Sample Trial Arm	
	Nutritional Supplement	Nutritional Supplement+Physical Activity	Physical Activity	Control	Nutritional Supplement	Control
**Health center characteristics**						
Number of centers per arm	5	5	5	5	4	4
Median (range) cohort in age range	961 (286–1,530)	819 (575–1,771)	938 (476–2,036)	664 (274–1,743)	1,228 (805–1,445)	1,338 (945–1,563)
Median percent (range) living in poverty^a^	9.6 (7.4–14.2)	8.0 (7.4–9.6)	8.6 (2.5–13.4)	10.8 (9.6–16.7)	8.7 (2.5–10.8)	8.7 (8–8.7)
**Participant characteristics**						
*n* b	502	516	480	504	400	397
Age (y)^c^	66.2 (0.9)	66.2 (1.0)	66.1(0.9)	66.1 (1.0)	66.5 (1.1)	66.6 (1.1)
*n* (%) male	160 (31.9)	163 (31.6)	141 (29.4)	186 (36.9)	124 (31.0)	132 (33.3)
**Level of education**						
*n* (%) 0–5 y schooling	146 (30.9)	138 (27.9)	111 (24.0)	161 (33.8)	155 (41.0)	100 (25.9)
*n* (%) 6–10 y schooling	232 (49.2)	271 (54.7)	241 (52.1)	241 (50.5)	180 (47.6)	168 (43.5)
*n* (%)>10 y schooling	94 (19.9)	86 (17.4)	111 (24.0)	75 (15.7)	43 (11.4)	118 (30.6)
***n*** ** (%) married or equivalent**	326 (64.9)	349 (67.6)	309 (64.4)	332 (65.9)	253 (63.3)	257 (64.7)
***n*** ** living in the house** c	3.7 (2.0)	3.8 (2.2)	4.0 (2.6)	3.7 (2.1)	3.9 (2.2)	3.7 (2.2)
***n*** ** (%) community participation**	165 (32.9)	187 (36.2)	187 (39.0)	162 (32.1)	n.d.	n.d.
***n*** ** (%) weekly physical activity**	47 (9.4)	41 (7.8)	48 (10.0)	34 (6.8)	37 (9.3)	47 (11.8)
**Self-reported health status**						
*n* (%) Good to excellent	221 (44.0)	221 (42.8)	226 (47.1)	196 (38.9)	159 (39.8)	211 (53.2)
*n* (%) Fair to poor	281 (56.0)	295 (57.2)	254 (52.9)	308 (61.1)	241 (60.2)	186 (46.8)
**MMSE short-form score** c	16.7 (2.0)	16.7 (1.9)	16.6 (2.3)	16.4 (2.1)	16.0 (2.3)	16.6 (2.0)
**GDS-15 score** d	2 (1–6)	2 (1–5)	2 (1–5)	2 (1–6)	n.d.	n.d.
*n* (%) scoring ≥5	153 (30.5)	164 (31.8)	141 (29.4)	159 (31.5)		
**SF-36** c						
Physical component score	49.9 (4.8)	50.0 (6.7)	51.2 (6.7)	49.8 (16.3)	n.d.	n.d.
Mental component score	49.6 (9.0)	49.3 (4.1)	49.3 (9.1)	49.4 (7.9)		
**Distance walked in 6 min (m)** c	455.3 (75.4)	452.4 (75.4)	444.4 (69.6)	450.7 (82.7)	n.d.	n.d.
**TUG (s)** c	10.0 (1.8)	9.7 (2.1)	10.0 (1.8)	10.1 (2.3)	n.d.	n.d.
**BMI (kg/m^2^)** d	28.2 (25.7–31.2)	28.3 (25.8–31.4)	28.4 (26.2–31.8)	28.6 (25.8–32.0)	28.6 (25.9–32.1)	28.4 (25.5–31.4)
**Weight (kg)** d	70.0 (61.0–78.5)	70.0 (61.5–77.3)	69.5 (62.0–78.7)	70.0 (62.5–79.0)	68.5 (61.0–77.4)	70.0 (62.8–77.0)
**Blood pressure (mmHg)**						
Systolic^c^	136.7 (19.7)	135.0 (20.1)	137.1 (20.1)	133.9 (18.6)	140.1 (20.7)	144.5 (21.1)
Diastolic^c^	81.1 (11.2)	80.2 (11.8)	81.1 (11.7)	79.2 (11.5)	82.1 (12.2)	85.2 (12.4)
*n* (%)≥140/90	198 (40.7)	193 (38.8)	183 (41.3)	162 (35.3)	191 (47.8)	233 (58.7)

a As defined by the 2006 census (per capita income less than twice the value of a standardized basic basket of food) [57].

b Represents total sample per trial arm at baseline. There were small amounts of missing data (maximum five participants per outcome); data available but not shown.

c Mean (standard deviation).

d Median (interquartile range).

GDS-15, 15-item Geriatric Depression Scale; MMSE, Mini Mental State Examination; n.d., no data, more limited information was collected for the supplementary sample; SF-36, 36-item Short-Form health survey. Scores calculated using an algorithm derived for Chilean older people [58].

Participants collected 68% of total available food, and adherence was 75% (73% in the nutritional supplement+physical activity arm, 77% in the original nutritional supplement only arm, and 78% in the four supplementary clusters receiving the nutritional supplement). Participants attended 24% of physical activity sessions offered, and adherence was 43% (47% in the nutritional supplement + physical activity arm and 38% in the physical activity only arm).

**Figure 2:**
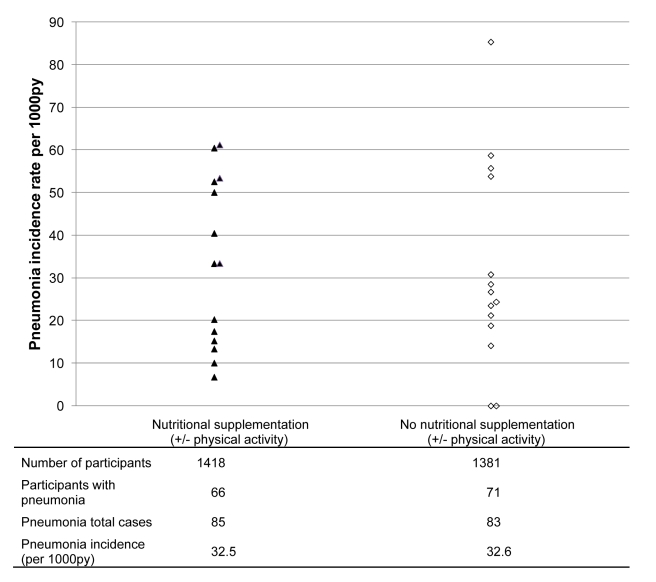
Pneumonia incidence rates per 1,000 py by cluster over 24 mo after enrolment based on 14 clusters in each trial arm. Closed triangles, nutritional supplement arm (nutritional supplement±physical activity); open diamonds, control arm (no nutritional supplement±physical activity).

Over 24 mo there were 85 cases of pneumonia in 1,418 participants in the 14 nutritional supplement intervention clusters and 83 cases in 1,381 participants in the 14 no nutritional supplement control clusters, equivalent to incidence rates of 32.5 versus 32.6 per 1,000 py respectively (risk ratio [RR] = 1.00; 95% CI 0.61–1.63; *p* = 0.99). Rates varied considerably across study clusters (CV = 0.51; ICC = 0.01) (Figure 2). Neither the data collected over the 6 mo after the end of intervention (15 cases) nor per-protocol analysis (RR = 1.05; 95% CI 0.65–1.69; *p* = 0.85) (Table S4) materially altered these results.

**Figure 3:**
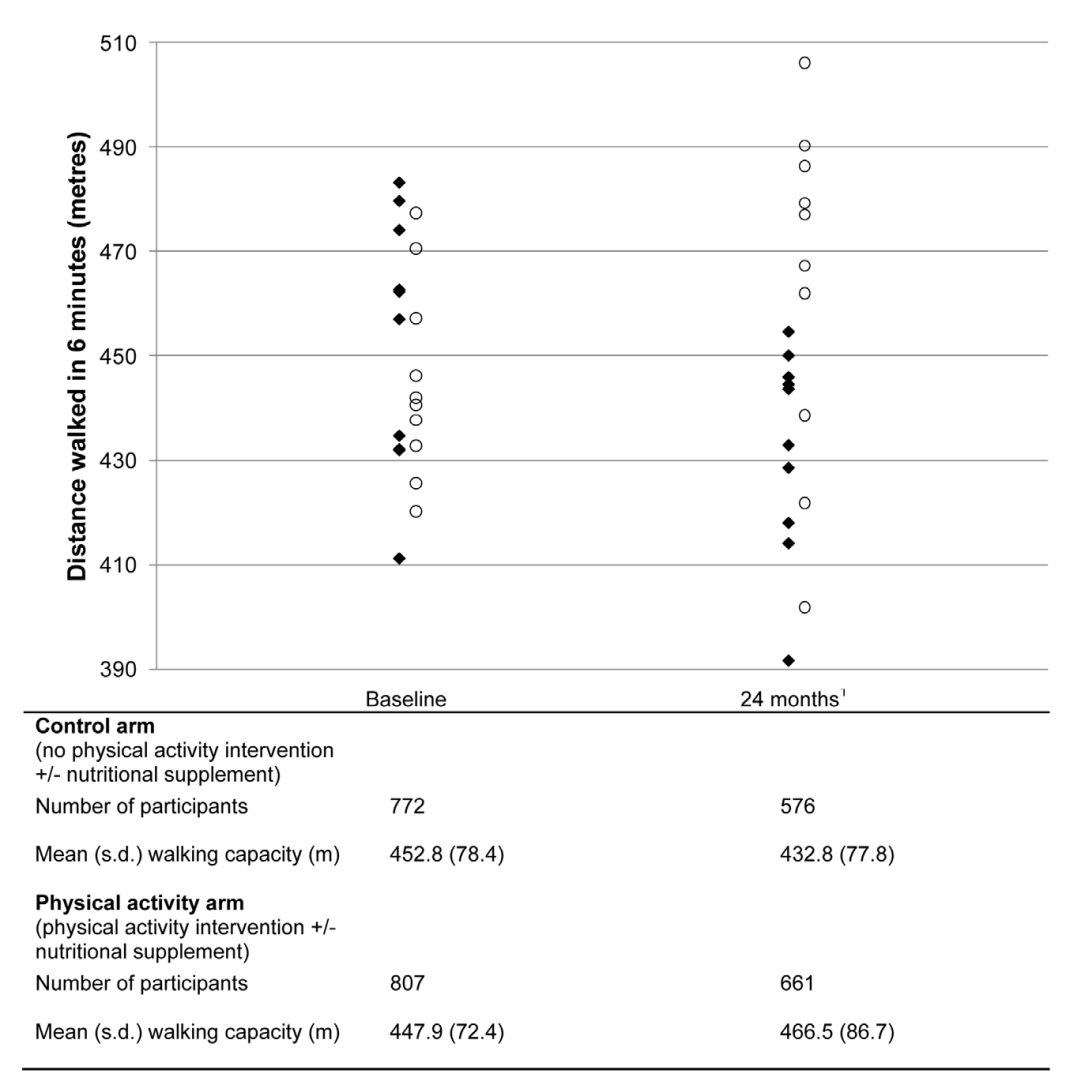
Walking capacity (distance walked in meters in 6 min) at 0 and 24 mo by cluster based on ten clusters in each trial arm. Open circles, physical activity arm (physical activity±nutritional supplement); closed diamonds, control arm (no physical activity intervention±nutritional supplement). ^1^Includes 20 participants in control arm and 24 participants in intervention arm who did not provide data at baseline.

The mean distance walked in 6 min, 24 mo after enrolment increased by 14.4 m in the ten physical activity intervention clusters and declined by 22.5 m in the ten no physical activity clusters, an estimated mean difference adjusted for clustering of 33.8 m (95% CI 13.9–53.8; *p* = 0.001; ICC = 0.09) (Figure 3). Per protocol analysis revealed a 54.4-m mean difference in walking capacity at 24 mo (95% CI 32.8–75.9; *p*<0.001). A significantly nonlinear (*p* = 0.005) dose response relationship was present between the change in walking capacity over 24 mo and the number of physical activity classes attended (Figure S1) and on average, participants walked 0.7 m further in 6 min at 24 mo for each additional exercise class attended. The effect of additional exercise classes on walking capacity increased significantly with each extra class attended (*p*<0.001).

Other than for the TUG test where the mean time was 0.54 s less in the physical activity intervention arms than the no physical activity control arms (95% CI −0.93 to −0.15; *p* = 0.006), none of the prespecified secondary outcome measures showed statistically significant effects of the interventions (Table 2). The RR for combined pneumonia and ALRI incidence was 1.08 (95% CI 0.82–1.41; *p* = 0.60) (Table S4). No serious adverse events were reported for either intervention and there was no difference between trial arms in self-reported falls or fractures. As specified in the protocol, we tested for an interaction between the two interventions for the BMI outcome, and we found no significant interaction between arms (*p* = 0.56; ICC = 0.08). BMI and weight declined slightly and nonsignificantly, and blood pressure increased slightly and nonsignificantly over 24 mo in all arms.

**Table 2:** Secondary outcomes at 24 mo in the 20 original clusters.

Variable	Trial arm			
	Nutritional Supplement	Nutritional Supplement+Physical Activity	Physical Activity	Control
*n* centers per arm	5	5	5	5
*n* participants^a^	414	452	403	400
**GDS-15 score** b	2 (1–6)	2 (1–7)	2 (1–5)	2 (1–7)
*n* (%) scoring ≥5	142 (34.3)	149 (32.9)	108 (26.8)	133 (33.3)
Change from 0 m^b^	0 (−1 to 2)	0 (−1 to 2)	0 (−2 to 1)	0 (−1 to 2)
**SF-36**, physical component score^c^	50.8 (6.3)	50.2 (8.3)	51.1 (14.3)	50.6 (8.9)
Change from 0 m^c^	0.9 (5.8)	0.2 (3.9)	−0.1 (1.1)	0.7 (11.6)
**SF-36**, mental component score^c^	48.9 (7.4)	49.2 (9.1)	49.2 (6.3)	48.3 (6.3)
Change from 0 m^c^	−0.7 (2.9)	−0.1 (7.3)	−0.1 (5.4)	−1.0 (6.4)
**TUG (s)** c	10.5 (2.5)	9.7 (3.0)	9.9 (2.5)	10.4 (2.1)
Change from 0 m^c^	0.4 (2.2)	−0.1 (2.6)	−0.1 (2.1)	0.3 (1.9)
**BMI (kg/m^2^)** b	28.2 (25.8–31.5)	28.1 (25.7–30.8)	28.5 (26.3–32.0)	28.2 (25.5–32.3)
Change from 0 m^b^	0.0 (−1.0, 1.0)	−0.2 (−1.3 to 0.7)	−0.1 (−1.2 to 0.9)	−0.5 (−1.3 to 0.7)
**Weight (kg)** b	69.0 (60.5–77.4)	68.0 (60.9–76.0)	69.0 (62.0–78.0)	69.8 (61.0–78.0)
Change from 0 m^b^	−1.0 (−3.0, 1.1)	−1.0 (−3.5 to 1.0)	−0.8 (−3.0 to 1.5)	−1.0 (−3.5 to 1.0)
**Blood pressure (mmHg)**				
Systolic^c^	139.4 (19.9)	135.5 (20.0)	139.4 (19.7)	135.4 (19.3)
Change from 0 m^c^	2.8 (19.8)	0.6 (20.1)	3.0 (19.7)	2.2 (17.7)
Diastolic^c^	81.3 (12.3)	78.7 (12.1)	80.1 (11.4)	79.7 (11.7)
Change from 0 m^c^	0.2 (11.9)	−1.5 (11.8)	−0.5 (11.1)	1.1 (10.7)
*n* (%)≥140/90	195 (47.1)	178 (39.4)	191 (47.4)	154 (38.5)
***n*** ** (%) falls and fractures**				
Missing	89	64	78	106
Falls	191 (46)	213 (47)	189 (47)	198 (50)
Fractures (wrist only)^d^	8 (2)	6 (1)	10 (2)	5 (1)

a Represents total sample per trial arm at 24 mo. There were small amounts of missing data (maximum five participants per outcome); data available but not shown.

b Median (interquartile range).

c Mean (standard deviation).

d No reported hip fractures.

GDS-15, 15-item Geriatric Depression Scale; SF-36, 36-item Short-Form health survey. Scores calculated using an algorithm derived for Chilean older people [58].

The overall costs over 24 mo were US$91.00 and US$163.70 per participant for the nutritional supplement and physical activity interventions, respectively. The cost of the physical activity intervention per extra meter walked at 24 mo was US$4.84, or US$3.01, based on the per protocol analysis.

## Discussion

This is, to our knowledge, the first randomized trial conducted in a transitional country setting designed to determine the cost-effectiveness of nutritional supplement and physical activity interventions among older people. We recruited 2,799 participants aged 65.0–67.9 y from 28 health centers in low-middle socioeconomic municipalities in Santiago, Chile, and retained 84% of study participants over 24 mo of intervention. The results of the trial do not support the hypothesis of benefit, in terms of prevention of pneumonia, from micronutrient supplementation at the doses provided in Chile's ongoing national nutritional supplementation program for older people. The provision of physical activity classes improved physical function, assessed by a 34-m increase in walking capacity (approximately 8%) in adults aged 65–67 y, at a cost of US$4.84 per additional meter walked.

The strengths of this study include its large size, randomized design, and high level of participant retention. The willingness of the Chilean Ministry of Health to support the study provides important precedents for future evaluations and the potential for consequent evidence-based policy.

There are also several weaknesses in the study. First, because of the design, the interventions were not masked to participants. However, physicians diagnosing the primary outcome for the nutritional intervention were not formally involved in the study nor were they aware of participants' involvement in the study or the interventions being provided. Similarly, fieldworkers collecting information on physical activity outcomes did not know the intervention being provided to particular participants or their compliance with the exercise regimen. Second, although this was a large study, the lack of effect detected on incidence of pneumonia might be partly due to insufficient statistical power. The revised sample size for the pneumonia outcome was based on incidence data over the first 4 mo of the study, but the observed coefficient of variation was considerably higher than originally estimated (0.51 versus 0.14). While a certain degree of intercluster variability in pneumonia rates is to be expected, the absolute size of this variability has not previously been assessed in Chile at the health center level. Furthermore, the study was conducted at a time when pneumonia rates were decreasing, and access to influenza immunization programs increasing, both of which factors might have influenced the intercluster variability. Also, our results could not exclude the possibility that a micronutrient supplementation program may benefit other measures of health (physical or mental) in older people. We are similarly not able to make any comment on the possible effect of the interventions on those individuals excluded at study entry. Third, recruitment of participants within the physical activity arms was slightly lower than in the other arms suggesting that participants may have been less motivated to join a study in which they were offered an exercise intervention. There were no obvious differences in the characteristics of the groups at baseline. Loss to follow up was slightly higher in the control arm, possibly reflecting fewer intervention-related contacts. Fourth, while adherence to the nutritional supplementation intervention was generally high and in accordance with national data, which suggest that more than 85% of individuals who receive these supplements from their health center report that they consume it on a regular basis [40], adherence to the physical activity intervention was relatively moderate, despite attempts to make the classes easily accessible and enjoyable. The enhanced effect on walking capacity among those adults with higher adherence suggests that greater adherence to the study protocol may have increased the size of the effect. And finally, the restricted and (relative to very elderly) youthful age range of the study participants, while appropriate for our study rationale, may have limited the potential impact of the interventions.

This is the first large trial to investigate the effect of micronutrient supplements for the prevention of infection in older people outside high-income settings [10],[11], to our knowledge, and the lack of any detected effect of supplementation extends the evidence base to include transition economies. The doses of the various micronutrients provided in the nutritional supplementation are similar to those provided in other published trials [10], and there is currently no evidence that larger safe doses would have a different effect. Previous trials on benefits of resistance training for older people have been small, brief, and to date have only been conducted in developed countries generally under fully supervised conditions. A recent Cochrane review comparing resistance training with a no-exercise control in older people (11 trials included; total in analysis *n* = 325; mean age 63–84 y) found that resistance training resulted in a statistically significant 52-m increase in walking capacity over 6 min [12]. The review also estimated (12 trials included; total in analysis *n* = 691, mean age 66–85 y) that resistance training resulted in a statistically significant 0.7-s decrease in TUG [12]. We detected a 34-m benefit of physical activity classes on walking capacity and a 0.5-s benefit in the TUG test. Our findings extend the results from the Cochrane review in two important ways. First, the physical activity intervention was made available in local community halls and there was no intensive follow-up of study participants. Second, this is the first study to be conducted outside a high-income country.

A Cochrane review of interventions to reduce falls in older people living in the community identified 17 trials that compared exercise interventions with no-exercise control [41]. Overall, the trials suggested that exercise interventions reduced the risk of falls (RR 0.83; 95% CI 0.72–0.97). This review was based on small (*n* = 28–437; total in analysis = 2,494) and brief (5–52 wk) trials, only one of which was set outside a high-income country. Results from our study suggest that the physical activity intervention resulted in a nonsignificant reduction in the risk of falls (RR 0.98; 95% CI 0.89–1.07) and thus do not support the conclusion from the Cochrane review. However, it is important to note that the sample recruited into the current study had a more restricted age range than that of some other studies, which may partially explain the discrepancy between the current finding and the existing literature.

Simple measures of walking speed and ability have been widely used as markers of functional ability in later life. Walking speed has repeatedly been shown to be a powerful predictor of health-related events and mortality in healthy older people [42]–[46]. The evidence base for the 6-min walk relates mostly to individuals with preexisting respiratory problems, but similarly suggests that poor performance on the test is predictive of mortality [47],[48]. Indeed, a 6-min walk of less than 350 m is one of four components of a prediction score for death in patients with chronic obstructive pulmonary disease (COPD) [49]. In a study of 112 individuals with stable COPD, Redelmeier and colleagues [50] estimated a difference of 54 m (95% CI 37–74) in 6-min walk distances was associated with a noticeable difference in patients' subjective comparison ratings of their walking ability and was clinically relevant [51],[52]. There are no guidelines for clinical relevance of the 6-min walk in healthy older people; however, enhanced walking capacity as demonstrated in this study is directly correlated with improved walking speed and, thus, it may be associated with improved health or quality of life outcomes, especially among populations who walk as their primary means of transport.

Given the intervention-specific nature of the numerator (additional meters walked), it is not possible to compare the relative cost-effectiveness of physical activity classes to other health care interventions in Chile and Latin America. Economic evaluations of similar physical activity programs from other settings suggest that it may represent value for money. For example, a review of health care–based interventions aimed at improving physical activity identified five controlled trials in older populations, all of which demonstrated favorable cost-effectiveness [53]. A more recent cluster randomized trial of a community based exercise program in ≥65-y-olds in the UK demonstrated that it cost approximately €17,000 to gain a quality-adjusted life year (QALY), which again compares favorably with other health care interventions [54]. We estimate that rolling out the exercise program to all older people in Chile would account for approximately 5% of the government's US$3.3 billion health care expenditure, although we would expect that such scaling up would substantially reduce unit and thus total costs. A community-based intervention of the kind provided in the CENEX study, which is practical, affordable, and enjoyable for participants, provides further evidence to support the contention that exercise is a “best buy”‘ in public health terms [55].

It is important to recognize that there may be broader, long-term economic effects on the population or health service that have not been accounted for in the instruments used to assess economic components. For example, wider benefits may include social capital created through the physical activity intervention, and an increase in the level of trust in health services and participants' willingness to use such services, which, in turn, may influence the effectiveness of future programs.

Chile has a high burden of infectious and nutrition-related chronic diseases, which impose significantly on the national health budget, thus increasing the importance of identifying cost-effective preventive public health interventions [56]. From the results of this study, there is little evidence to support the effectiveness of Chile's national nutritional supplementation program in reducing the incidence of pneumonia for 65.0–67.9-y-olds. However, the provision of physical activity classes to older people, especially at high levels of adherence, may well have clinical benefit for older people in metropolitan settings such as Santiago. Future challenges include increasing uptake to, and retention in, sustainable physical activities, and exploring mechanisms for extending the benefit of physical activity both beyond metropolitan Santiago and into other age groups.

## Supporting Information

Figure S1 Dose response curve of change in distance walked in 6 min (meters) after 24 mo of intervention against number of physical activity classes attended by study participants from clusters randomized to physical activity intervention.
(PDF)

Table S1 Nutritional composition (per 100 g) of the food supplements (Años Dorados and Bebida Láctea) provided in the CENEX study.
(PDF)

Table S2 Baseline characteristics of all 28 clusters in the nutritional supplement versus no nutritional supplement <1?show=[to]?>comparison.
(PDF)

Table S3 Baseline characteristics of all 20 clusters in the physical activity versus no physical activity comparison.
(PDF)

Table S4 Secondary analysis of pneumonia outcomes in CENEX study of adults aged 65–67 y in Santiago, Chile: per protocol analysis of adherent individuals (A); analysis of combined pneumonia and ALRI incidence in total study sample over 24 mo (B).
(PDF)

Text S1 Consort checklist.
(DOCX)

Text S2 CENEX study protocol [22].
(PDF)

Text S3 CENEX study cost-effectiveness protocol [33].
(PDF)

## Abbreviations

ALRI: acute lower respiratory infection
BMI: body mass index
CENEX: Cost-effectiveness Evaluation of a Nutritional supplement and EXercise program for older people
CI: confidence interval
CV: coefficient of variation
ICC: intracluster coefficient
PACAM: Programme of Complementary Feeding for the Older Population
RR: risk ratio
TUG: timed up-and-go
