# Supplementary material for: Effect of a Nutrition Supplement and Physical Activity Program on Pneumonia and Walking Capacity in Chilean Older People: A Factorial Cluster Randomized Trial
Source: PLoS Med. 2011 Apr 19;8(4):e1001023. doi: 10.1371/journal.pmed.1001023 (PMC3079648; doi:10.1371/journal.pmed.1001023)
Supplement: Figure S1 — Dose response curve of change in distance walked in 6 min (meters) after 24 mo of intervention against number of physical activity classes attended by study participants from clusters randomized to physical activity intervention. (PDF) [file pmed.1001023.s001.pdf]

Figure S1*: Dose response curve of change in distance walked in 6 minutes (meters) after 24 months of intervention against number of physical activity classes attended by study participants from clusters randomized to physical activity intervention*[a,b]

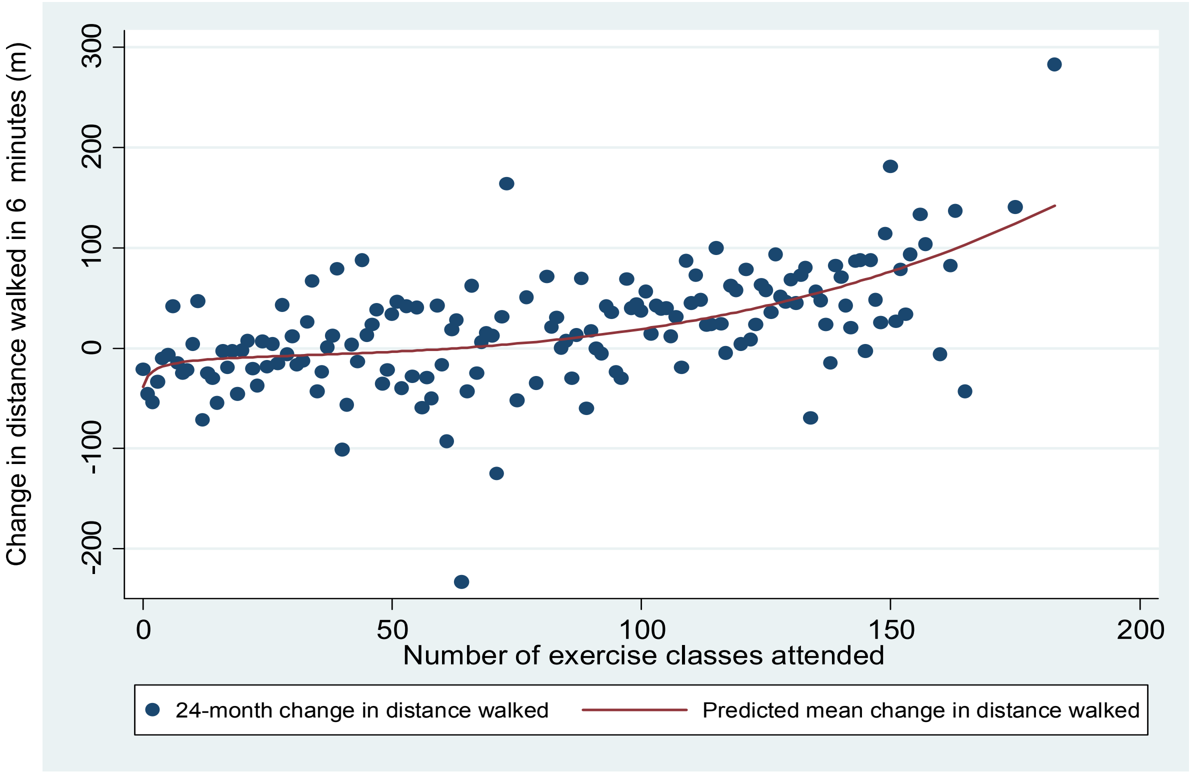

[a]Figure represents data from 637 participants. Individual dots may represent more than one participant.

[b]Shape of curve not material altered by removal of “outlier” at >250m
