# Supplementary material for: Effect of a Nutrition Supplement and Physical Activity Program on Pneumonia and Walking Capacity in Chilean Older People: A Factorial Cluster Randomized Trial
Source: PLoS Med. 2011 Apr 19;8(4):e1001023. doi: 10.1371/journal.pmed.1001023 (PMC3079648; doi:10.1371/journal.pmed.1001023)
Supplement: Table S1 — Nutritional composition (per 100 g) of the food supplements (Años Dorados and Bebida Láctea) provided in the CENEX study. (PDF) [file pmed.1001023.s002.pdf]

Table S1*: Nutritional composition (per 100g) of the food supplements (Años Dorados and Bebida Láctea) provided in the CENEX study*

	**Años Dorados** per 100g	**Bebida Láctea** per 100g
Energy (kcal)	400	406
Protein (g)	13.0	18.0
Total fat (g)	11.0	10.0
Carbohydrates (g)	62.3	61.0
Total fiber (g)	6.0	1.0
**Vitamins/minerals**		
Vitamin A (μg RE)	240.0	800.0
Vitamin C (mg)	30.0	180.0
Vitamin D (μg)	8.0	16.0
Vitamin E (mg TE)	16.0	32.0
Vitamin B_1 (mg)	0.4	0.8
Vitamin B_2 (mg)	0.4	1.6
Niacin (mg NE)	4.5	10.0
Vitamin B_6 (mg)	1.0	1.6
Folate (μg)	100.0	400.0
Vitamin B_{12} (μg)	1.4	2.8
Sodium (mg)	280.0	540.0
Calcium (mg)	400.0	1000.0
Iron (mg)	4.2	5.6
Phosphorus (mg)	400.0	800.0
Magnesium (mg)	150.0	300.0
Zinc (mg)	3.0	12.0

RE, retinol equivalents; TE, alpha-tocopherol equivalents; NE, niacin equivalents
