# Supplementary material for: Effect of a Nutrition Supplement and Physical Activity Program on Pneumonia and Walking Capacity in Chilean Older People: A Factorial Cluster Randomized Trial
Source: PLoS Med. 2011 Apr 19;8(4):e1001023. doi: 10.1371/journal.pmed.1001023 (PMC3079648; doi:10.1371/journal.pmed.1001023)
Supplement: Table S2 — Baseline characteristics of all 28 clusters in the nutritional supplement versus no nutritional supplement <1?show=[to]?>comparison. (PDF) [file pmed.1001023.s003.pdf]

Table S2*: Baseline characteristics of all 28 clusters in the nutritional supplement vs. no nutritional supplement comparison*

Variable	Trial arm<br>Nutritional supplement (+/- physical activity intervention)	 No nutritional supplement (+/- physical activity intervention)
**Health centre characteristics**		
Number of centers per arm	14	14
Median (range) cohort in age-range	1065 (286, 1530)	1017 (274, 2036)
Median % (range) living in poverty[a]	8.5 (2.5, 14.2)	9.2 (2.5, 16.7)
**Participant characteristics**		
n[b]	1418	1381
Age (years)[c]	66.2 (1.0)	66.2 (1.0)
n (%) male	447 (31.5)	459 (33.2)
**Level of education**		
n (%) 0-5 years schooling	439 (32.6)	372 (28.1)
n (%) 6-10 years schooling	683 (50.8)	650 (49.0)
n (%) >10 years schooling	223 (16.6)	304 (22.9)
n (%) **married or equivalent**	928 (64.4)	898 (65.0)
n **living in house**[c]	3.8 (2.1)	3.8 (2.2)
n (%) **weekly physical activity**	125 (8.8)	129 (9.3)
**Self-reported health status**		
n (%) Good to excellent	601 (42.4)	633 (45.8)
n (%) Fair to poor	817 (57.6)	748 (54.2)
**MMSE short-form score**[c]	16.5 (2.0)	16.5 (2.1)
**Body Mass Index (kg/m^2)**[d]	28.3 (25.8,31.5)	28.5 (25.7,31.8)
**Weight (kg)**[d]	69.7 (61,78)	69.8 (62,78)
**Blood pressure (mmHg)**		
Systolic[c]	137.0 (20.2)	138.3 (20.4)
Diastolic[c]	81.1 (11.7)	81.7 (12.1)
n (%) $\geq$140/90	582 (42.2)	578 (44.5)

[a]As defined by the 2006 census (per capita income less than twice the value of a standardized basic basket of food) [57]

[b]Represents total sample per trial arm at baseline. There were small amounts of missing data (maximum 5 participants per outcome) – data available but not shown

[c]Mean (standard deviation)

[d]Median (inter-quartile range)

MMSE, Mini Mental State Examination
