# Supplementary material for: Effect of a Nutrition Supplement and Physical Activity Program on Pneumonia and Walking Capacity in Chilean Older People: A Factorial Cluster Randomized Trial
Source: PLoS Med. 2011 Apr 19;8(4):e1001023. doi: 10.1371/journal.pmed.1001023 (PMC3079648; doi:10.1371/journal.pmed.1001023)
Supplement: Table S3 — Baseline characteristics of all 20 clusters in the physical activity versus no physical activity comparison. (PDF) [file pmed.1001023.s004.pdf]

Table S3: *Baseline characteristics of all 20 clusters in the physical activity vs. no physical activity comparison*

Variable	Trial arm<br>Physical activity (+/- nutritional supplement intervention)	 No physical activity (+/- nutritional supplement intervention)
**Health centre characteristics**		
Number of centers per arm	10	10
Median (range) cohort in age-range	878.5 (476, 2036)	848 (274, 1743)
Median % (range) living in poverty[a]	8.3 (2.5, 13.4)	10.2 (7.4, 16.7)
**Participant characteristics**		
n[b]	996	1006
Age (years)[c]	66.2 (0.9)	66.1 (0.9)
n (%) male	304 (30.5)	346 (34.4)
**Level of education**		
n (%) 0-5 years schooling	249 (26.0)	307 (32.3)
n (%) 6-10 years schooling	512 (53.4)	473 (49.8)
n (%) >10 years schooling	197 (20.6)	169 (17.8)
n (%) **married or equivalent**	658 (66.1)	658 (65.4)
n **living in the house**[c]	3.9 (2.4)	3.7 (2.0)
n (%) c**ommunity participation**	374 (37.6)	327 (32.5)
n (%) **weekly physical activity**	89 (8.9)	81 (8.1)
**Self-reported health status**		
n (%) Good to excellent	447 (44.9)	417 (41.4)
n (%) Fair to poor	549 (55.1)	589 (58.6)
**MMSE short-form score**[c]	16.7 (2.1)	16.5 (2.1)
**GDS-15 score**[d]	2 (1, 5)	2 (1, 6)
n (%) scoring ≥5	305 (30.8)	312 (31.5)
**SF-36**[c]		
Physical component score	50.6 (6.7)	49.8 (12.0)
Mental component score	49.3 (7.0)	49.4 (8.5)
**Distance walked in 6 minutes (m)**[c]	448.6 (72.7)	453.0 (78.6)
**Timed up-and-go (seconds)**[c]	9.8 (2.0)	10.0 (2.1)
**Body Mass Index (kg/m^2)**[c]	28.3 (26.0, 31.6)	28.4 (25.8, 31.6)
**Weight (kg)**[d]	69.8 (62.0, 78.0)	70.0 (62, 79)
**Blood pressure (mmHg)**		
Systolic[c]	136.0 (20.1)	135.4 (19.2)
Diastolic[c]	80.6 (11.8)	80.2 (11.4)
n (%) ≥140/90	376 (40.0)	360 (38.1)

[a]As defined by the 2006 census (per capita income less than twice the value of a standardized basic basket of food) [57]

[b]Represents total sample per trial arm at baseline. There were small amounts of missing data (maximum 5 participants per outcome) – data available but not shown

[c]Mean (standard deviation)

[d]Median (inter-quartile range)

MMSE, Mini Mental State Examination; GDS-15, 15-item Geriatric Depression Scale; SF-36,36-item Short-Form health survey. Scores calculated using an algorithm derived for Chilean older people [58]
