# Supplementary material for: Effect of a Nutrition Supplement and Physical Activity Program on Pneumonia and Walking Capacity in Chilean Older People: A Factorial Cluster Randomized Trial
Source: PLoS Med. 2011 Apr 19;8(4):e1001023. doi: 10.1371/journal.pmed.1001023 (PMC3079648; doi:10.1371/journal.pmed.1001023)
Supplement: Table S4 — Secondary analysis of pneumonia outcomes in CENEX study of adults aged 65–67 y in Santiago, Chile: per protocol analysis of adherent individuals (A); analysis of combined pneumonia and ALRI incidence in total study sample over 24 mo (B). (PDF) [file pmed.1001023.s005.pdf]

Table S4*: Secondary analysis of pneumonia outcomes in CENEX study of adults aged 65-67 years in Santiago, Chile: per protocol analysis of adherent individuals (top panel A); analysis of combined pneumonia and acute lower respiratory infection (ALRI) incidence in total study sample over 24 months (bottom panel B)*

**Variable**	**Nutritional supplement (+/- physical activity**	**No nutritional supplement (+/- physical activity)**	**Adjusted RR (95% CI)**	**Statistic**	**p-value**
**A. Per protocol analysis for pneumonia primary outcome**					
Number of health centers	14	14			
Number of participants	1080	1381			
Participants with pneumonia	53	71			
Pneumonia total cases	68	83			
Pneumonia incidence (per 1000 PY)	34.1	32.6	1.05 (0.65-1.69)	Z=0.18	0.85
**B. Combined pneumonia and ALRI incidence rates**					
Number of health centers	14	14			
Number of participants	1418	1381			
Participants with pneumonia or ALRI	261	227			
Pneumonia or ALRI cases	388	352			
Pneumonia or ALRI incidence (per 1000 PY)	148.9	139.0	1.08 (0.82-1.41)	Z=0.54	0.59
