# Supplementary material for: Effect of a Nutrition Supplement and Physical Activity Program on Pneumonia and Walking Capacity in Chilean Older People: A Factorial Cluster Randomized Trial
Source: PLoS Med. 2011 Apr 19;8(4):e1001023. doi: 10.1371/journal.pmed.1001023 (PMC3079648; doi:10.1371/journal.pmed.1001023)
Supplement: Text S1 — Consort checklist. (DOCX) [file pmed.1001023.s006.docx]

CONSORT 2010 checklist of information to include when reporting a cluster randomised trial

| \|  \| \| \| \|  \| \| --- \| --- \| --- \| --- \| --- \| \| **Section/Topic** \| **Item No** \| **Checklist item** \|  \| **PAGE** \| \| **Title and abstract** \| \| \| \|  \| \|  \| 1a \| Identification as a cluster randomised trial in the title \|  \| TITLE \| \| 1b \| Structured summary of trial design, methods, results, and conclusions (for specific guidance see CONSORT for abstracts^21 31^) \|  \| 2 \| \| **Introduction** \| \| \| \|  \| \| Background and objectives \| 2a \| Scientific background and explanation of rationale, including rationale for using a cluster design \|  \| 3/4 \| \| 2b \| Specific objectives or hypotheses and whether they pertain to the individual level or the cluster level or both \|  \| 4 \| \| **Methods** \| \| \| \|  \| \| Trial design \| 3a \| Description of trial design (ie cluster and any additional design features such as parallel, factorial) including allocation ratio \|  \| 4 \| \| 3b \| Important changes to methods after trial commencement (such as eligibility criteria), with reasons \|  \| 5/6 \| \| Participants \| 4a \| Eligibility criteria for clusters and individual participants \|  \| 5/6/7 \| \| 4b \| Settings and locations where the data were collected \|  \| 5 \| \| Interventions \| 5 \| The interventions for each group, whether they pertain to the individual level or the cluster level or both, with sufficient details to allow replication, including how and when they were actually administered \|  \| 5 \| \| Outcomes \| 6a \| Completely defined pre-specified primary and secondary outcome measures, whether they pertain to the individual level or the cluster level or both, including how and when they were assessed \|  \| 6/8/9 \| \| 6b \| Any changes to trial outcomes after the trial commenced, with reasons \|  \| NA \| \| Sample size \| 7a \| How total sample size was determined (including method of calculation, number of clusters, cluster size, a coefficient of intracluster correlation (ICC or k), and an indication of its uncertainty) \|  \| 7/8 \| \| 7b \| When applicable, explanation of any interim analyses and stopping guidelines \|  \| 9 \| \| Randomisation: \|  \|  \|  \|  \| \| Sequence generation \| 8a \| Method used to generate the random allocation sequence \|  \| 6 \| \| 8b \| Type of randomisation; details of any restriction such as blocking (and block size) , stratification, matching \|  \| 6 \| \| Allocation concealment mechanism \| 9 \| Mechanism used to implement the random allocation sequence (such as sequentially numbered containers), specifying that allocation was based on clusters rather than individuals and describing any steps taken to conceal the sequence until interventions were assigned \|  \| 6 \| \| Implementation \| 10 \| Who generated the random allocation sequence, who enrolled clusters and individual participants, and who assigned clusters to interventions \|  \| 6 \| \| Blinding \| 11a \| If done, who was blinded after assignment to interventions (for example, participants, care providers, those assessing outcomes) and how \|  \| 8/9/12 \| \| 11b \| If relevant, description of the similarity of interventions \|  \| NA \| \| Statistical methods \| 12a \| Statistical methods used to compare groups for primary and secondary outcomes indicating how clustering was taken into account \|  \| 9/10 \| \| 12b \| Methods for additional analyses, such as subgroup analyses and adjusted analyses. \|  \| NA \| \| **Results** \| \| \| \|  \| \| Cluster and individual participant flow (a diagram is strongly recommended) \| 13a \| For each group, the numbers of clusters and individual participants who were randomly assigned, received intended treatment, and were analysed for the primary outcome Describe protocol deviations from study as planned, together with reasons. \|  \| Figure 1  Page 7 \| \| 13b \| For each group, losses and exclusions after randomisation, together with reasons \|  \| Figure 1 \| \| Recruitment \| 14a \| Dates defining the periods of recruitment and follow-up \|  \| 6/9 \| \| 14b \| Why the trial ended or was stopped \|  \| NA \| \| Baseline data \| 15 \| A table showing baseline demographic and clinical characteristics for each group for the individual and cluster levels as applicable \|  \| Tables 1/S2/S3 \| \| Numbers analysed \| 16 \| For each group, number of clusters and individual participants (denominators) included in each analysis and whether the analysis was by original assigned groups \|  \| Page 9  Figures 1/2/3  Table S4 \| \| Outcomes and estimation \| 17a \| For each primary and secondary outcome, results for each group (the individual or cluster level as applicable), and the estimated effect size and its precision (such as 95% confidence interval) and a coefficient of intracluster correlation (ICC or k) for each primary outcome. \|  \| Page 9/10  Figure 2/3  Tables 2/S4 \| \| 17b \| For binary outcomes, presentation of both absolute and relative effect sizes is recommended \|  \| NA \| \| Ancillary analyses \| 18 \| Results of any other analyses performed, including subgroup analyses and adjusted analyses, distinguishing pre-specified from exploratory \|  \| NA \| \| Harms \| 19 \| All important harms or unintended effects in each group (for specific guidance see CONSORT for harms^28^) \|  \| Page 11  Table 2 \| \| **Discussion** \| \| \| \|  \| \| Limitations \| 20 \| Trial limitations, addressing sources of potential bias, imprecision, and, if relevant, multiplicity of analyses \|  \| 12/13 \| \| Generalisability \| 21 \| Generalisability (external validity, applicability) of the trial findings to individuals and/or clusters (as relevant) \|  \| 13/14/15 \| \| Interpretation \| 22 \| Interpretation consistent with results, balancing benefits and harms, and considering other relevant evidence \|  \| 13/14/15 \| \| **Other information** \| \| \|  \|  \| \| Registration \| 23 \| Registration number and name of trial registry \|  \| 1 \| \| Protocol \| 24 \| Where the full trial protocol can be accessed, if available \|  \| Refs. 22/33 \| \| Funding \| 25 \| Sources of funding and other support (such as supply of drugs), role of funders \|  \| Suppl. information \| |
| --- | --- | --- | --- | --- | --- | --- | --- | --- | --- | --- | --- | --- | --- | --- | --- | --- | --- | --- | --- | --- | --- | --- | --- | --- | --- | --- | --- | --- | --- | --- | --- | --- | --- | --- | --- | --- | --- | --- | --- | --- | --- | --- | --- | --- | --- | --- | --- | --- | --- | --- | --- | --- | --- | --- | --- | --- | --- | --- | --- | --- | --- | --- | --- | --- | --- | --- | --- | --- | --- | --- | --- | --- | --- | --- | --- | --- | --- | --- | --- | --- | --- | --- | --- | --- | --- | --- | --- | --- | --- | --- | --- | --- | --- | --- | --- | --- | --- | --- | --- | --- | --- | --- | --- | --- | --- | --- | --- | --- | --- | --- | --- | --- | --- | --- | --- | --- | --- | --- | --- | --- | --- | --- | --- | --- | --- | --- | --- | --- | --- | --- | --- | --- | --- | --- | --- | --- | --- | --- | --- | --- | --- | --- | --- | --- | --- | --- | --- | --- | --- | --- | --- | --- | --- | --- | --- | --- | --- | --- | --- | --- | --- | --- | --- | --- | --- | --- | --- | --- | --- | --- | --- | --- | --- | --- | --- | --- | --- | --- | --- | --- | --- | --- | --- | --- | --- | --- | --- | --- | --- | --- | --- | --- | --- | --- | --- | --- | --- | --- | --- | --- | --- | --- | --- | --- | --- | --- | --- | --- | --- | --- | --- | --- | --- | --- | --- | --- | --- | --- |
