# Supplementary material for: Effect of a Nutrition Supplement and Physical Activity Program on Pneumonia and Walking Capacity in Chilean Older People: A Factorial Cluster Randomized Trial
Source: PLoS Med. 2011 Apr 19;8(4):e1001023. doi: 10.1371/journal.pmed.1001023 (PMC3079648; doi:10.1371/journal.pmed.1001023)
Supplement: Text S3 — CENEX study cost-effectiveness protocol [33]. (PDF) [file pmed.1001023.s008.pdf]

BMC Health Services Research

Study protocol

Open Access

Methods for economic evaluation of a factorial-design cluster randomised controlled trial of a nutrition supplement and an exercise programme among healthy older people living in Santiago, Chile: the CENEX study

Damian G Walker*[1], Cristian Aedo[2], Cecilia Albala[3], Elizabeth Allen[4], Alan D Dangour[5], Diana Elbourne[4], Emily Grundy[6] and Ricardo Uauy[5]

Address: [1]Department of International Health, Bloomberg School of Public Health, Johns Hopkins University, Baltimore, USA, [2]Human Development Department, Latin America and the Caribbean Region, The World Bank, 1818 H Street, NW Washington, DC 20433, USA , [3]Instituto de Nutrición y Tecnología de los Alimentos (INTA), University of Chile, Santiago, Chile, [4]Medical Statistics Unit, Department of Epidemiology and Population Health, London School of Hygiene & Tropical Medicine (LSHTM), London, UK, [5]Nutrition and Public Health Intervention Research Unit, Department of Epidemiology and Population Health, London School of Hygiene & Tropical Medicine, London, UK and [6]Centre for Population Studies, Department of Epidemiology and Population Health, London School of Hygiene & Tropical Medicine, London, UK

Email: Damian G Walker* - dgwalker@jhsph.edu; Cristian Aedo - caedo@worldbank.org; Cecilia Albala - calbala@uchile.cl; Elizabeth Allen - Elizabeth.Allen@lshtm.ac.uk; Alan D Dangour - Alan.Dangour@lshtm.ac.uk; Diana Elbourne - Diana.Elbourne@lshtm.ac.uk; Emily Grundy - Emily.Grundy@lshtm.ac.uk; Ricardo Uauy - Ricardo.Uauy@lshtm.ac.uk

* Corresponding author

Published: 27 May 2009

BMC Health Services Research 2009, **9**:85 doi:10.1186/1472-6963-9-85

Received: 10 April 2009
Accepted: 27 May 2009

This article is available from: http://www.biomedcentral.com/1472-6963/9/85

© 2009 Walker et al; licensee BioMed Central Ltd.
This is an Open Access article distributed under the terms of the Creative Commons Attribution License (http://creativecommons.org/licenses/by/2.0), which permits unrestricted use, distribution, and reproduction in any medium, provided the original work is properly cited.

Abstract

Background: In an effort to promote healthy ageing and preserve health and function, the government of Chile has formulated a package of actions into the Programme for Complementary Food in Older People (Programa de Alimentación Complementaria para el Adulto Mayor - PACAM). The CENEX study was designed to evaluate the impact, cost and cost-effectiveness of the PACAM and a specially designed exercise programme on pneumonia incidence, walking capacity and body mass index in healthy older people living in low- to medium-socio-economic status areas of Santiago. The purpose of this paper is to describe in detail the methods that will be used to estimate the incremental costs and cost-effectiveness of the interventions.

Methods and design: The base-case analysis will adopt a societal perspective, including the direct medical and non-medical costs borne by the government and patients. The cost of the interventions will be calculated by the ingredients approach, in which the total quantities of goods and services actually employed in applying the interventions will be estimated, and multiplied by their respective unit prices. Relevant information on costs of interventions will be obtained mainly from administrative records. The costs borne by patients will be collected via exit and telephone interviews. An annual discount rate of 8% will be used, consistent with the rate recommended by the Government of Chile. All costs will be converted from Chilean Peso to US dollars with the 2007 average period exchange rate of US$1 = 522.37 Chilean Peso. To test the robustness of model results, we will vary the assumptions over a plausible range in sensitivity analyses.

Discussion: The protocol described here indicates our intent to conduct an economic evaluation alongside the CENEX study. It provides a detailed and transparent statement of planned data collection methods and analyses.

Trial registration: ISRCTN48153354

Background

The United Nations Population Division has estimated great increases in both the proportion and absolute size of the older person population over the next fifty years [1]. At the 2nd World Assembly on Ageing, policy initiatives were proposed to promote active and healthy lifestyles in later life [2]. Emphasis was placed, among other things, on adequate nutrition throughout the life course and national food policies designed to recognise older people as potentially vulnerable. The aim was to increase healthy life-years in older people, and thereby decrease the number of years, and the proportion of time, spent in poor health. Some governments have responded to these increased demands by designing innovative programmes aimed at tackling the twin problems of food and nutrition insecurity among older people [3]. For example, food baskets are still popular in some parts of Latin America, and community kitchens have also been experimented with, particularly in Argentina. In Mexico City, the local government provides a universal pension transferred electronically to a plastic debit card which can be used in supermarkets.

Chile is currently undergoing a period of rapid demographic transition which has led to an increase in the proportion of older people in the population. Many older people in Chile are socially and nutritionally vulnerable, and this has placed great demands on the national health care system which has limited human resources and budget. To promote healthy ageing, the Government of Chile has initiated a programme of nutritional supplementation which may have considerable positive benefits for the health and function of older people.

The overall aim of the Programme for Complementary Food in Older People (*Programa de Alimentación Complementaria para el Adulto Mayor* – PACAM) is to contribute to improvements in health and quality of life among older people in Chile. In order to be eligible for the programme, individuals must be registered at their local health centre and be 70+ years of age.

Chile's approach to nutrition and health care has been criticised as being too expensive for most low- and middle-income countries [3], and no information is currently available on the cost-effectiveness of the supplementation programme. Therefore there is interest in evaluating the cost-effectiveness of PACAM. At the same time, there have also been calls in Chile to enhance the performance of PACAM by including a physical activity component [4].

Here we present a summary of economic evaluation methods being applied in the CENEX study [ISRCTN48153354]. The design of the main study is described in detail elsewhere [4]. Briefly, as the nutritional supplements are distributed to older people at health centres, and the exercise programme is also offered to groups, the study was designed as a 2 × 2 factorial cluster-randomised controlled trial of the effect of a two year intervention. The interventions consisted of either a nutritional supplement, or an exercise programme, or both, or neither. The primary outcomes are the incidence of pneumonia and walking capacity among adults aged 65.0–67.9 years at baseline living in low- to medium-socio-economic status areas of Santiago, Chile.

Methods and design

Economics is gaining increasing importance in health care practice and research. Economic evaluation methods provide a framework to inform programme planners about the viable options for intervention, and the levels at which they would best be implemented, taking into account the objectives of the intervention. This study has been designed to include an economic evaluation alongside a cluster-randomised controlled trial.

Economic evaluation requires specification of

- the frames of the study;
- the interventions;
- the health outcomes;
- the 'economic' outcomes;
- data presentation and analysis; and
- sensitivity analysis.

Framing the economic evaluation

The first step to conducting an economic evaluation is to frame the study. Decisions made at this stage directly determine which costs and outcomes are considered relevant and should therefore be included in the analysis. This means that choices made in the framing of the evaluation will have an impact on the final results of an analysis.

The target audience includes all persons or institutions that will use the results of the study to make decisions. For this study the primary target audience is the Chilean Ministry of Health. Secondary audiences include older Chilean adults themselves, as well as policy-makers and older adults in other relevant settings.

The study question of an economic evaluation should be well-defined, stated in an answerable form and relevant to the decision facing the target audience. For this study the study question is two-fold: (i) can nutritional supplementation and physical exercise prevent disease and functional disability in later life, i.e. do they work?, and (ii) if so, are they worth it?, i.e. do they represent an efficient use

of resources? The focus of this paper is on the methods to assess the efficiency, rather than the effectiveness, of the interventions.

The choice of perspective, or viewpoint, determines the scope of the costs and benefits. For this study we will adopt a health system and societal perspective. The target population of this study is participants aged 65.0–67.9 years at baseline who lived within the defined catchment area of the selected health centres. The time frame for the CENEX trial is two years. This does not allow the full long-term cost and benefits to be measured. Therefore, some modelling is planned and is described in more detail below with respect to the calculation of health outcomes.

What are the interventions?

There are two components of the nutrition intervention within PACAM: one is a powdered food called Golden Years (*Años Dorados*), which is composed of a cereal and legume mix fortified with vitamins and minerals; the other called *Bebida Láctea* is a similarly fortified low-lactose powdered milk. Every beneficiary is entitled to collect a one-kilo sachets of each product from their local health post, health centre or hospital each month.

For the exercise intervention, participants are individually invited to attend two physical activity training sessions per week of one hour each. Training consists of a period of warming up and three levels of chair stands, three levels of modified squats, three levels of step-ups onto a stair, and six sets of 15 repetitions of arm pull-ups using rubber bands of variable resistance. Special exercises are delivered to participants with specific physical limitations. Participants are encouraged to walk to the exercise classes (up to 20 minutes from home) as part of the exercise protocol. Participants unable to attend classes are advised about exercises that could be conducted in their homes.

What are the health outcomes?

The primary outcome for the nutrition intervention (PACAM) will be the incidence of pneumonia over a two-year period after the initiation of the intervention. The study is using the following operational definitions of pneumonia:

1. Hospitalised cases of pneumonia – J10-J12 (viral) and J13-J18 (bacterial);

2. Health centre diagnosed and treated pneumonia.

The primary outcome for the exercise intervention will be walking capacity (distance walked in six minutes) assessed two years after initiation of the intervention.

The following secondary outcomes will be considered:

- Body mass index [BMI weight (kg)/height (m)^2] as a measure of potential interaction between the two interventions;

- Measure of self-reported health status (Short-Form 36);

- Measure of depression (GDS-15);

- Self-reported incidence of chronic diseases (diabetes, hypertension, coronary heart disease, stroke, heart failure) from baseline to 24 months after the initiation of the intervention;

- Self reported productive activity in the household and community;

- Self-reported incidence of falls from baseline to 24 months after the initiation of the intervention;

- Self-reported incidence of fracture from baseline to 24 months after the initiation of the intervention;

- Blood pressure;

- Anthropometry (waist circumference, triceps skin-fold thickness, mid-arm circumference, calf circumference, hand-grip strength;

- Timed up-and-go assessment;

- Blood indicators of cardiovascular disease risk and insulin resistance (in a sub-sample).

In addition to these measures of self-reported health status, morbidity and function, we will also consider the impact of the interventions on reducing mortality. We will use evidence on the association between walking capacity (distance and speed) and mortality risk to extrapolate our intermediate endpoints (distance walked in 6 minutes and timed up-and-go assessment) to final outcomes. Walking speed represents a powerful predictor of health-related events in older persons [5]. Ostir et al. [6] illustrated that walking speed alone can provide similar information on mortality risk to a more comprehensive summary measure of physical performance. Finally, we plan to calculate quality-adjusted life-years (QALYs) using self-reported health-related quality of life data collected with the SF-36 instrument. Estimates of lifetime QALYs will be based on predicted life expectancy using age- and gender-specific period mortality rates for the population of Santiago, Chile.

What are the 'economic' outcomes

The following section provides a description of the methods for estimating the costs of providing and accessing the

interventions. These costs will also be supplemented by the costs of treating the disease(s) the intervention(s) aims to avoid and the additional health care the intervention(s) might trigger. These data will be collected over the intervention period using a range of methods.

Patient costs will be assessed via:

- exit interviews of study participants to ascertain the costs of accessing the interventions (PACAM and exercise classes). A questionnaire will be administered by local interviewers, and includes closed questions (with options for 'no response' and 'don't know') asking how the participants travelled to the health facility (to collect the supplement or to receive care) or the exercise classes, the cost of their travel, how much time they spent both travelling and at the facility/class. To estimate opportunity costs, we will also ask about what they would have been doing otherwise, including lost earnings (if employed);

- case-based sampling in which individuals who have experienced episodes of pneumonia and acute respiratory infections will be identified, contacted and interviewed by telephone, in order to estimate the costs borne by them, and any caregivers. In the interviews, information will be collected on both hospitalised and non-hospitalised patients, including: transportation costs borne by the patient and carer(s); costs of care, number of days out of work (or other productive unpaid activities), income lost by patient and carer(s) (if employed).

The opportunity cost of the participants' or patients' time will be valued in monetary units, using a range of different approaches. These data will not be combined with out-of-pocket expenses. There is not a consensus among economists on a single appropriate valuation method. For example, we will use an estimate based on stated losses of each group, valued at average wage rates for people aged 65–67 years of age. We will use our data on activities that would have been carried out if they were not at the health centre or exercise class, and try to obtain appropriate shadow wage rates where these activities are non-remunerated.

Provider costs will be assessed via:

- defining the types, quantities and prices of the different components required to deliver the interventions (PACAM and exercise classes) at the health centres and/or community centres by reviewing expenditure and budget records. Given that the major set-up costs of the national primary health infrastructure, through which PACAM is delivered, are already covered, the additional cost of implementing PACAM corresponds to the incremental cost associated with the distribution of the two nutritional supplements and the associated staff time. Indeed, sufficient staff time may already exist. The annual equivalents of all capital costs will be calculated on the basis of lifetimes appropriate to each item. An annual discount rate of 8% will be used, consistent with the rate recommended by the Government of Chile.

- case-based sampling, in which the medical records of individuals who have experienced episodes of pneumonia and acute lower respiratory infections will be identified, information will be collected on both hospitalised and non-hospitalised patients, including: length of stay; number of emergency room visits; drugs consumed and tests performed.

- analysis of existing data on health centre utilisation rates will be used to measure differences in utilisation between the trial arms, and we will identify unit costs associated with the different services provided or averted. The costs of providing these services, e.g. diagnoses and treatment of the secondary outcome variables such as diabetes, hypertension, coronary heart disease, stroke, heart failure and fractures, will be estimated based on the Ministry of Health reference costs (available on the website of AUGE – *Acceso Universal para prestaciones integrales y Garantías Explícitas asociadas a la atención de prioridades*). Because full analysis of the costs of these secondary outcome variables is beyond the scope of the current study, the user costs for these services will be modelled from data collected on pneumonia and acute lower respiratory infection cases.

All costs will be converted from Chilean Peso to US dollars with the 2007 average period exchange rate of US$1 = 522.37 Chilean Peso. All setup costs and other costs related to the CENEX study will be excluded; these include costs associated with trial administration, data collection, and actual measurement of clinical outcomes as per the trial protocol. Because no previous costing studies could be called upon to give a sense of the expected variation, we did not conduct a formal power calculation to determine the sample size for the data collection activities. Instead, the size of samples required for the collection of these data will be calculated once the overall variability of the key measures is determined – to this end, we plan to begin with the preliminary samples sizes indicated in Table 1.

Data presentation and analysis

Costs falling upon the health sector, patients or their families will be presented in total and disaggregated form. Resource use and unit costs will be used to estimate mean,

medians, standard deviations and ranges of costs for the samples of participants in the CENEX study.

We will consider two types of economic evaluation. If there is some evidence of clinical differences between interventions, the incremental costs and effects between the interventions will be combined to estimate the incremental cost-effectiveness of one intervention over another, i.e. a cost-effectiveness analysis (CEA). This will identify the cost associated with a given change in health effect represented in terms of an incremental cost-effectiveness ratio (ICER). If one intervention is both more effective and less costly than the other, and if there are no other unmeasured factors which would alter choices, then it is dominant and should be adopted widely. If one intervention is more effective and more costly than the other, then the results will inform the extra cost to society of an increase in a specified health gain.

Additionally, we will consider cost-utility analysis (CUA) where consequences are measured in term of utility (QALYs). This form of evaluation reflects quantity and quality of life changes as a result of the interventions in a single composite measure. We will reflect on the potential bias of using utility measures such as the QALY since we require reliable and valid measures for application to older population groups that can also be used within the context and for the purpose of economic evaluation. For example, what is the impact of age on assessments of patient-focused health status (including patient-reported outcomes) and health-related quality of life (HRQOL)? Do existing measures inherently bias cost-effectiveness results against the particular group of older people (due to older patients being less likely to benefit for long periods of time from an intervention compared to younger age groups)? Does the QALY adequately represent the health gains experienced by older people? For example, Donaldson et al. [7] described QALYs as being inappropriate for evaluating long-term care for older people due to its insensitivity to changes in health status within this context.

Table 1: Summary of the preliminary sample sizes

Patient costs	
Cost of accessing PACAM	100 exit interviews
Cost of accessing the exercise programme	100 exit interviews
Cost of hospitalised pneumonia	10 telephone interviews
Cost of ambulatory pneumonia	25 telephone interviews
Cost of ambulatory pneumonia	25 telephone interviews
Provider costs	
Cost of hospitalised pneumonia	10 medical records
Cost of ambulatory pneumonia	25 medical records
Cost of ambulatory ARI	25 medical records

In economic evaluations of health care interventions to date, many analysts have simply presented results without interpretation or recommendation; furthermore, definitions of the threshold, or ceiling ratio, have been left largely to the discretion of the analyst. The ceiling ratio represents a decision maker's valuation of a unit of health gain and is a particularly crucial and politically sensitive element of economic evaluation as it is the relative value against which the acceptability of ICERs are judged. If the value of an ICER is below the ceiling ratio, an intervention is deemed acceptable on grounds of cost-effectiveness. Based on the recommendation of the Commission on Macroeconomics and Health (CMH) [8], WHO classifies interventions as 'highly cost-effective' for a given country if results show that they gain a QALY for less than the per capita national GNI or GDP. In 2007, GNI per capita in Chile was estimated to be $8,350. We will also place the findings in broader context by comparing them to other economic evaluations that have been undertaken in Chile or neighbouring countries after adjustments have been made for inflation.

Before any conclusions are made, any assumptions and uncertainties will be tested using sensitivity analysis. Sensitivity analysis will help in the evaluation of the reliability of the conclusions for the particular context of the trial and will also facilitate consideration of the generalisability of results within and beyond Chile.

It would be beneficial to health care decision makers if economic study results could be generalised from one setting to another as this would avoid having to repeat every study in every setting. Factors which may vary in different settings are: access to care, unit costs of resources, geographical variations in demography or epidemiology of disease, clinical practice patterns and quality of care, and availability of resources. To facilitate estimation of the transferability of economic data from the CENEX study to other health care settings both within and beyond Chile, such factors in the study population will be described, and resource use and prices reported separately.

Ethical approval

The protocol for this study has been approved by the Institutional Review Board at Instituto de Nutrición y Tecnología de los Alimentos, University of Chile, and by the London School of Hygiene and Tropical Medicine ethics committee.

Discussion

The CENEX study and associated economic evaluation provide a unique opportunity to measure the costs and cost-effectiveness of a nutrition supplement and an exercise programme prospectively, alongside the actual health care processes for which effectiveness is being measured.

In this paper, the methods have been described for evaluating whether these interventions will be cost-effective. Presenting this methodology paper before the end of the trial makes transparent the methods used for the evaluation. In our view it is important to record our methods in detail before publication of the results of the trial so that a record of detail not normally found in the final trial reports can be made available in the public domain.

There may be broader, long-term economic effects on the population or health service that, because of the scope of the CENEX study, have not been accounted for in the instruments used to assess the clinical and economic components. For example, wider benefits may include the value attached by the participants to the process of receiving such services. Indeed a number of studies have shown that in low- and middle-income countries, older people are less respected and less valued than their healthier counterparts [9,10]. Benefits may also derive from institutional change. Institutions, in this sense, are defined as the patterns of behaviour that determine how individuals, groups and organisations interact with one another [11] and may be either informal (e.g. various norms of behavior) or formal (legislation, government policy, regulations). Institutions are relevant to programmes for older people since they are defined by specific relationships – in particular those they establish between providers and clients (and the wider community). The development of such relationships, to some extent, alters the nature of the community itself e.g. they may increase the level of trust in health services and individuals' willingness to use such services which, in turn, may influence the effectiveness of future programmes. Holistic approaches to carrying out economic evaluation using institutionalist methods to account for some of these broader issues have recently been examined [12], although these themes have generally not been well recognised in economic evaluation.

Incorporation of economic evaluations within (cluster) randomised controlled trials of public health interventions has been a growing trend in the past decade [13,14]. Many health care systems now use economic evaluations as a formal input to decisions about whether to fund programmes. Although there is an expressed need for economic evaluation in Latin America, very few examples of the use of such studies exist. A survey conducted by Iglesias et al. [15] showed that decisions regarding the allocation of resources to different areas of health care in Latin America are mainly driven by governmental policies. Furthermore, the lack of a clearly defined set of criteria to facilitate or guide the decision-making processes within the Latin American health-care systems was identified as one of the main obstacles preventing the use of economic evaluation studies. Given that this study is supported by the Ministry of Health in Chile, we are confident that it will have an influence on policy and practice.

Conclusion

Unprecedented changes in the demographic characteristics of countries worldwide mean that we are all confronted by the challenge of preparing to meet the demands of an ageing society. In the face of current demographic trends, increasing health care costs, and concerns about the quality of health care, the financing and delivery of care for older people is a critical health care policy challenge. A focused research effort to determine how health care systems can most cost-effectively prevent disability, reduce functional decline, and extend active life expectancy in older people such as that described here for the CENEX study will provide decision-makers with the information needed to tackle age-specific disability rates and to allocate limited resources efficiently. These methods should also be of use for the economic evaluation of similar nutrition and exercise programmes.

Competing interests

The authors declare that they have no competing interests.

Authors' contributions

AD and RU conceived the overall study. DW and CA designed the economic evaluation. CA, CA, AD, EG, RU and DW applied for funding. DE and LA provided cluster trial expertise. All authors read and approved the final protocol.

Acknowledgements

The funding for the CENEX study has been provided by The Wellcome Trust. The Ministry of Health, Chile has provided in kind support for the implementation of the study.

References

1. United Nations: *World Population Prospects, the 2000 revision, Comprehensive Tables Volume 1*. New York, United Nations; 2001.
2. WHO: *Active Ageing: A Policy Framework* World Health Organization, Geneva; 2002.
3. Dangour AD, Moreno X, Albala C, Rivera-Marquez A, Lera L, Villalobos A, Morris SS, Uauy R: **Chile's national nutritional supplementation program for older people: lessons learned.** *Food Nutr Bull* 2005, **26:**190-197.
4. Dangour AD, Albala C, Aedo C, Elbourne D, Grundy E, Walker D, Uauy R: **A factorial-design cluster randomised controlled trial investigating the cost-effectiveness of a nutrition supplement and an exercise programme on pneumonia incidence, walking capacity and body mass index in healthy older people living in Santiago, Chile: the CENEX study protocol [ISRCTN48153354].** *Nutrition Journal* 2007, **6(1):**14.
5. Cesari M, Kritchevsky SB, Penninx BW, Nicklas BJ, Simonsick EM, Newman AB, Tylavsky FA, Brach JS, Satterfield S, Bauer DC, Visser M, Rubin SM, Harris TB, Pahor M: **Prognostic value of usual gait speed in well-functioning older people – results from the Health, Aging and Body Composition Study.** *J Am Geriatr Soc* 2005, **53(10):**1675-80.
6. Ostir GV, Kuo YF, Berges IM, Markides KS, Ottenbacher KJ: **Measures of lower body function and risk of mortality over 7 years of follow-up.** *Am J Epidemiol* 2007, **166(5):**599-605.

7. Donaldson C, Atkinson A, Bond J, Wright K: **Should QALY's be programme specific?** *Journal of Health Economics* 1988, **7:**239-257.
8. Commission on Macroeconomics and Health: **Macroeconomics and health: investing in health for economic development.** Boston: Center for International Development at Harvard University; 2001.
9. Glascock AP, Fineman SL: **Social asset or social burden: treatment of the aged in non-industrial societies.** In *Ageing, Culture and Health* Edited by: Fry CL. JF Bergin Publishers, New York; 1981.
10. Guillette EA: **Change and continuity for older Tswana.** *J Cross-Cult Gerontol* 1990, **5:**191-204.
11. North DC: **Institutions and economic theory.** *American Economist* 1992, **36(1):**3-6.
12. Jan S, Pronyk P, Kim J: **Accounting for institutional change in health economic evaluation: a program to tackle HIV/AIDS and gender violence in Southern Africa.** *Soc Sci Med* 2008, **66(4):**922-32.
13. Goodacre S, Nicholl J, Dixon S, Cross E, Angelini K, Arnold J, Revill S, Locker T, Capewell SJ, Quinney D, Campbell S, Morris F: **Randomised controlled trial and economic evaluation of a chest pain observation unit compared with routine care.** *BMJ* 2004, **328(7434):**254.
14. Mortimer D, French SD, McKenzie JE, O'Connor DA, Green SE, the IMPLEMENT study group: **Protocol for economic evaluation alongside the IMPLEMENT cluster randomised controlled trial.** *Implement Sci* 2008, **3(1):**12.
15. Iglesias CP, Drummond MF, Rovira J, NEVALAT Project Group: **Health-care decision-making processes in Latin America: problems and prospects for the use of economic evaluation.** *Int J Technol Assess Health Care* 2005, **21(1):**1-14.

Pre-publication history

The pre-publication history for this paper can be accessed here:

http://www.biomedcentral.com/1472-6963/9/85/prepub

Publish with **BioMed Central** and every scientist can read your work free of charge

"BioMed Central will be the most significant development for disseminating the results of biomedical research in our lifetime."

Sir Paul Nurse, Cancer Research UK

Your research papers will be:

- available free of charge to the entire biomedical community
- peer reviewed and published immediately upon acceptance
- cited in PubMed and archived on PubMed Central
- yours — you keep the copyright

Submit your manuscript here:
http://www.biomedcentral.com/info/publishing_adv.asp

BioMedcentral
